# The broad-spectrum antibiotic, zeamine, kills the nematode worm *Caenorhabditis elegans*

**DOI:** 10.3389/fmicb.2015.00137

**Published:** 2015-02-26

**Authors:** Josephine E. E. U. Hellberg, Miguel A. Matilla, George P. C. Salmond

**Affiliations:** ^1^Department of Biochemistry, University of CambridgeCambridge, UK; ^2^Department of Environmental Protection, Estación Experimental del Zaidín – Consejo Superior de Investigaciones CientíficasGranada, Spain

**Keywords:** *Serratia plymuthica*, *Dickeya*, secondary metabolite, PKS, NRPS, antibiotic, *Caenorhabditis elegans*, zeamine

## Abstract

Soil bacteria can be prolific producers of secondary metabolites and other biologically active compounds of economic and clinical importance. These natural products are often synthesized by large multi-enzyme complexes such as polyketide synthases (PKSs) or non-ribosomal peptide synthases (NRPSs). The plant-associated Gram-negative bacterium, *Serratia plymuthica* A153, produces several secondary metabolites and is capable of killing the nematode worm *Caenorhabditis elegans*; a commonly used model for the study of bacterial virulence. In this study, we show that disruption of the hybrid PKS/NRPS zeamine (*zmn*) gene cluster results in the attenuation of “fast-killing” of *C. elegans*, indicating that zeamine has nematicidal activity. *C. elegans* also exhibits age-dependent susceptibility to zeamine, with younger worms being most sensitive to the bioactive molecule. The *zmn* gene cluster is widely distributed within *Serratia* and phytopathogenic *Dickeya* species and investigation of strains harboring the *zmn* gene cluster showed that several of them are highly virulent in *C. elegans*. Zeamine was described previously as a phytotoxin and broad-spectrum antibacterial compound. In addition to its nematicidal properties, we show here that zeamine can also kill *Saccharomyces cerevisiae* and *Schizosaccharomyces pombe*. The expression of the *zmn* gene cluster and regulation of zeamine production were also investigated. Transcription of the cluster was growth phase-dependent, and was modulated by the post-transcriptional RNA chaperone, Hfq. The results of this study show that zeamine is a highly toxic molecule with little, or no, apparent host specificity in very diverse biological systems. In its current form, zeamine(s) may be useful as a lead compound suitable for chemical modification and structure-activity assays. However, because of widespread non-selective toxicity in multiple bioassays, unmodified zeamine(s) is unlikely to be suitable as a therapeutic antibiotic.

## INTRODUCTION

Bacteria belonging to the Gram-negative genus *Serratia* are members of the γ-proteobacterial family *Enterobacteriaceae*. The best characterized member of the genus, *Serratia marcescens*, was first described in 1819 as a pigmented microbial isolate (Grimont and Grimont, 1978). *Serratia* species are ecologically diverse although strains of *Serratia plymuthica* are commonly isolated from soil as well as from the rhizosphere of wheat (Åström and Gerhardson, 1988), oilseed rape (Neupane et al., 2012a,b,c), melon (Kamensky et al., 2003), and pea (Gould et al., 2008), the anthosphere of oil pumpkin (Fürnkranz et al., 2012) and from rotting potato tissue (Czajkowski et al., 2012). Many strains of *S. plymuthica* are capable of producing compounds with antibiotic activity, such as the antifungal and anti-oomycete haterumalide, oocydin A (Levenfors et al., 2004), and the antifungal pyrrolnitrin (Liu et al., 2007). Several *S. plymuthica* strains were shown to be effective as biocontrol agents (De Vleesschauwer and Höfte, 2003), for example being capable of controlling the phytopathogenic gray mold *Botrytis cinerea* and white mold, *Sclerotina sclerotiorum,* on greenhouse-grown melon (Kamensky et al., 2003), as well as suppressing the growth of *Penicillium* blue and green mold on citrus fruit (Meziane et al., 2006). The strain used in this study, *S. plymuthica* A153, was isolated from the rhizosphere of wheat (Åström and Gerhardson, 1988), despite producing phytopathogen-antagonistic compounds (Thaning et al., 2001; Levenfors et al., 2004). Recently, the strain has also been shown to be virulent in the *Caenorhabditis elegans* animal infection model (Matilla et al., 2012).

*Caenorhabditis elegans* is a free-living nematode that reaches an adult length of 1–2 mm. Although first developed as a genetic model in the 1970s (Brenner, 1974), *C. elegans* has undergone a renaissance over the past decade as a model system for assaying and understanding bacterial pathogenesis (Sifri et al., 2005). The importance of this has been two-fold. Firstly, not only are some soil-dwelling nematodes agricultural pests that attack economically important crops (reviewed by Jones et al., 2013) but they are also hard to control with traditional pesticides, and that makes nematode-pathogenic bacteria attractive as potential biocontrol agents (Chitwood, 2002). Secondly, although nematodes and mammals are separated by over 900 million years of evolution (Hedges et al., 2006), there are a surprising number of bacterial virulence factors affecting both taxa (Rahme et al., 2000). Altogether, *C. elegans* has become a useful model for the identification of novel virulence factors and the characterization of the relationships between pathogens and genetically amenable hosts (Sifri et al., 2005).

Bacteria are capable of antagonizing *C. elegans* by several mechanisms (Sifri et al., 2005). The most common of these is bacterial colonization of the nematode intestine, where bacteria accumulate in the intestinal lumen (causing it to expand) and interfere with its normal function (Sifri et al., 2005). This is the mechanism by which many human pathogens – such as *Salmonella typhimurium* (Aballay et al., 2000), *Pseudomonas aeruginosa* (Mahajan-Miklos et al., 1999), and *S. marcescens* (Kurz et al., 2003) – infect *C. elegans*. This type of ‘worm killing,’ also referred to as ‘slow killing,’ takes place over the span of several days and correlates with bacterial proliferation in the intestine (Sifri et al., 2005; Portal-Celhay et al., 2012). On the other hand, some bacteria are capable of killing worms over much shorter time-spans. This ‘fast killing’ is usually mediated by toxins that are produced by the bacteria (Sifri et al., 2005). Although toxic proteins have been described (Wei et al., 2003), the toxins are typically secondary metabolites. For example, *P. aeruginosa* PA14 is capable of killing *C. elegans* through oxidative stress by using the phenazine compound pyocyanin as a virulence factor (Mahajan-Miklos et al., 1999; Cezairliyan et al., 2013).

Secondary metabolites are traditionally considered to be non-essential organic molecules that are synthesized by cells during the later stages of growth, without playing any direct role in growth or development (Price-Whelan et al., 2006). Although the true roles of secondary metabolites remain elusive, some of them can confer fitness advantages to producing bacteria. Thus, secondary metabolites with antibiotic activity are thought to increase the fitness of bacteria in complex natural environments by antagonizing microbial competitors (Mazzola et al., 1992) or by deterring predation (Pradel et al., 2007). Additionally, since soil is a stressful environment where nutrients are limited (Challis and Hopwood, 2003), it has been speculated that soil bacteria are enriched for the production of secondary metabolites performing primary functions acting, for example, as synergistic siderophores (Price-Whelan et al., 2006). Many secondary metabolites are synthesized by large multidomain proteins such as non-ribosomal peptide synthases (NRPSs) or polyketide synthases (PKSs) (Sattely et al., 2008). Frequently, the genes encoding these enzymes are carried on the biosynthetic gene clusters together with the genes for tailoring enzymes responsible for the modification of the final structure of the molecule (Osbourn, 2010). The modular and mobile nature of biosynthetic gene clusters can enable the mixed assembly of biosynthetic genes from different sources to form hybrid gene clusters containing both NRPS- and PKS-encoding genes.

Preliminary work in this laboratory showed that *S. plymuthica* A153 is a nematode-pathogen capable of killing *C. elegans* rapidly (Matilla et al., 2012), suggesting that it produced a nematicide toxin. In this study we investigated the relationship between A153 and *C. elegans* to characterize the pathogen-worm interaction, with particular emphasis on identifying the A153 nematicide, genes involved in its biosynthesis and the regulation thereof.

## MATERIALS AND METHODS

### STRAINS, PLASMIDS, PHAGES, CULTURE MEDIA, AND GROWTH CONDITIONS

Bacterial strains, plasmids, and phages used in this study are listed in **Table 1**. *Serratia, Dickeya*, and derived strains were grown at 30°C, unless otherwise indicated, in L broth (LB, per liter: 5 g yeast extract, 10 g tryptone, 5 g NaCl), potato dextrose (16 g of potato dextrose broth l^-1^), minimal medium [MM: 0.1% (w/v) (NH_4_)_2_SO_4_, 0.41 mM MgSO_4_, 15 mM carbon source, 40 mM K_2_HPO_4_, 14.7 mM KH_2_PO_4_, pH 6.9–7.1], optimized minimal medium [OMM: 0.2% (w/v) (NH_4_)_2_SO_4_, 1.66 mM MgSO_4_, 0.2% (w/v) mannitol, 0.2% (w/v) glycerol, 60.3 mM K_2_HPO_4_, 33.1 mM KH_2_PO_4_, 15.9 μM MnCl_2_, 90.1 μM CaCl_2_, 32.9 μM FeSO_4_], 1-carbon OMM [1C-OMM: 0.2% (w/v) (NH_4_)_2_SO_4_, 1.66 mM MgSO_4_, 15 mM carbon source, 60.3 mM K_2_HPO_4_, 33.1 mM KH_2_PO_4_, 15.9 μM MnCl_2_, 90.1 μM CaCl_2_, 32.9 μM FeSO_4_]. *Escherichia coli* strains were grown at 37°C in LB. *E. coli* DH5α was used for gene cloning. Media for propagation of *E. coli* β2163 was supplemented with 300 μM 2,6-diaminopimelic acid. Where appropriate, antibiotics were used at the following final concentrations (in μg mL^-1^): ampicillin, 100; kanamycin, 25 (*E. coli*), 75 (*Serratia*); streptomycin, 50; tetracycline, 10. Sucrose was added to a final concentration of 10% (w/v) to select for derivatives of a second crossover event during marker exchange mutagenesis. *C. elegans* was maintained at 15°C using standard methods (Brenner, 1974). *Saccharomyces cerevisiae* and *Schizosaccharomyces pombe* were grown at 30°C in yeast peptone dextrose (YPD, per liter: 10 g yeast extract, 20 g peptone, 20 g glucose).

**Table 1:** Strains, phages, and plasmids used in this study.

Strain	Genotype or relevant characteristic^a^	Reference or source
***Serratia plymuthica* A153 strains**
A153	Wild-type, rhizosphere isolate (Zea^+^)	Åström and Gerhardson (1988)
A153L^b^	*lac-* derivative of A153	Matilla and Salmond (unpublished results)
A153A	*lac-,*Δ*andR,* made by marker exchange mutagenesis	Matilla and Salmond (unpublished results)
A153C	*lac-,*Δ*csrB*	Matilla and Salmond (unpublished results)
A153H	*lac-,*Δ*hfq,* made by marker exchange mutagenesis	Matilla and Salmond (unpublished results)
A153AH	*lac-,*Δ*andR*Δ*hfq,* made by marker exchange mutagenesis	Matilla and Salmond (unpublished results)
A153P	*lac-,*Δ*pigP*::Km, Km^r^	Matilla and Salmond (unpublished results)
A153R	*lac-,*Δ*rpoS*::Km, Km^r^	Matilla and Salmond (unpublished results)
A153T6	*lac-*; 1.259 bp deletion of the promoter region of the two predicted transcriptional units of the T6SS of A153; made by marker exchange mutagenesis	Matilla and Salmond (unpublished results)
A153Ce10	*lac-, zmn16*::mini-Tn*5*Sm/Sp, Zea^-^, Sm^r^	This study
A153Ce10A	*lac-,*Δ*andR, zmn16*::mini-Tn*5*Sm/Sp, Zea^-^, Sm^r^; derivative of A153A following transduction using ΦMAM1 grown on strain A153Ce10	This study
A153JH1	*lac-*, Δ*andR*, *zmn16*::Tn-KRCPN1, Zea^-^; Km^r^	This study
A153JH5	*lac-*, Δ*andR, zmn18/19*::Tn-KRCPN1, Zea^-^; Km^r^	This study
A153JH6	*lac-*, Δ*andR, zmn13*::Tn-KRCPN1*lacZ*, Zea^-^; Km^r^	This study
A153JH6H	*lac-*, Δ*andR,* Δ*hfq, zmn13*::Tn-KRCPN1*lacZ*, Zea^-^, Km^r^; derivative of A153AH following transduction using ΦMAM1 grown on strain A153JH6	This study
A153JH8	*lac-*, Δ*andR, ydhI*::Tn-KRCPN1, Zea^-^; Km^r^	This study
A153JH9^c^	*lac-*, Δ*andR mpg*::Tn-KRCPN1, Zea^-^; Km^r^	This study
A153JH10	*lac-*, Δ*andR, ydhJ*::Tn-KRCPN1, Zea^-^; Km^r^	This study
A153JH11^c^	*lac-*, Δ*andR, far*::Tn-KRCPN1, Zea^-^; Km^r^	This study
A153JH14	*lac-*, Δ*andR ydhI*::Tn-KRCPN1, Zea^-^; Km^r^	This study
A153JH23	*lac-*, Δ*andR ydhI*::Tn-KRCPN1, Zea^-^; Km^r^	This study
A153JH24	*lac-*, Δ*andR zmn10*::Tn-KRCPN1, Zea^-^; Km^r^	This study
A153JH27	*lac-*, Δ*andR zmn9*::Tn-KRCPN1, Zea^-^; Km^r^	This study
A153JH28	*lac-*, Δ*andR zmn10*::Tn-KRCPN1, Zea^-^; Km^r^	This study
***Serratia* strains**
*S. plymuthica* AS9	Wild-type	Neupane et al. (2012a)
*S. plymuthica* AS12	Wild-type	Neupane et al. (2012b)
*S. plymuthica* AS13	Wild-type	Neupane et al. (2012c)
***Escherichia coli* strains**
OP50	uracil auxotroph	Brenner (1974)
β2163	F^-^ RP4-2-Tc::Mu Δ*dapA*::(*erm-pir*), Km^r^	Demarre et al. (2005)
***Bacillus subtilis* strains**
JH642	*pheA1 trpC*2	Perego et al. (1989)
***Dickeya* strains**
*Dickeya* sp. MK7	Wild-type	Pritchard et al. (2013b)
*Dickeya solani* MK10	Wild-type	Pritchard et al. (2013a)
*Dickeya solani* MK16	Wild-type	Pritchard et al. (2013a)
*Dickeya solani* IPO 2222	Wild-type	Pritchard et al. (2013a)
*Dickeya sp.* NCPPB 3274	Wild-type	Pritchard et al. (2013b)
*Dickeya sp.* CSL RW 240	Wild-type	Pritchard et al. (2013a)
**Fungal strains**
*Verticillium dahliae* 5368	Wild-type, plant pathogen	J. Cooper
*Saccharomyces cerevisiae*	Wild-type	S. Oliver
*Schizosaccharomyces pombe*	Wild-type	J. Mata
***Caenorhabditis elegans***
DH26	*fer-15-*(*b26*)	*Caenorhabditi*s genetics center
**Bacteriophage**
ΦMAM1	Generalized transducing phage for *S. plymuthica* A153	Matilla and Salmond (2014)
**Plasmids**
pKRCPN1	Km^r^, Tc^r^; Derivative of pDS1028*uidA* with the *uidA* and *cat* genes replaced with ´*lacZ* and *km* genes	Roberts (2010)
pUT-mini-Tn*5*-Sm/Sp	Delivery plasmid for mini-Tn*5*Sm/Sp, Ap^r^, Sm^r^	de Lorenzo et al. (1990)
pTRB30	pQE-80L (Quiagen)-based expression vector, Ap^r^ replaced by Km^r^, Km^r^	T. Blower
pJEEUH13	*hfq* expression vector, Km^r^	This study

*^a^Ap, ampicillin; Km, kanamycin; Sm, streptomycin; Tc, tetracycline; Zmn, zeamine. ^b^Strain A153L is considered the A153 wild-type precursor of derivatives used in this study and is referred to as such throughout. ^c^Transposon insertions in these strains were in previously unlabelled genes, and have been named as follows: A153JH9, mannose-1-phosphate guanyltransferase (*mpg*); A153JH11, ferric aerobactin receptor (*far*)*.

### DNA TECHNIQUES, DNA MANIPULATION, AND BIOINFORMATICS ANALYSES

Plasmid DNA was isolated using the Anachem Keyprep plasmid DNA kit. Manufacturer’s instructions were followed for DNA digestion (New England Biolabs). The Anachem gel recovery kit was used to recover DNA from agarose gels. Ligation reactions and total DNA extraction were performed using standard protocols (Sambrook et al., 1989). Competent cells were prepared using calcium chloride and transformations were performed using standard protocols (Sambrook et al., 1989). PCR fragments for cloning were amplified using *Phusion*® high fidelity DNA polymerase (New England Biolabs), and all sequences were confirmed. DNA sequencing was performed at the University of Cambridge DNA Sequencing Facility on an Applied Biosystems 3730xl DNA analyzer. Genome comparison analyses were performed using the wgVISTA on-line tool (Frazer et al., 2004). Open reading frames (ORFs) in the zeamine (*zmn*) gene cluster were predicted using Glimmer 3.0 (Delcher et al., 1999). BLAST was used for functional gene assignment. Protein domain organization was identified using the NCBI conserved domains database (Marchler-Bauer et al., 2011).

### *IN VITRO* GROWTH KINETICS

The kinetics of bacterial growth (OD_600_) were measured with a Unicam Heλios spectrophotometer at 600 nm, using bacterial cultures grown in LB or OMM at 215 r.p.m. at 25°C, unless otherwise indicated. Three replicates were used for each condition and strain.

### RANDOM TRANSPOSON MUTAGENESIS, MUTANT SCREENING, AND GENERALIZED TRANSDUCTION

Random transposon mutagenesis of A153 was performed by conjugation with *E. coli* β2163 harboring plasmids containing transposons Tn-KRCPN1 or mini-Tn*5*Sm/Sp, using a previously published protocol (Matilla et al., 2012). Mutant libraries for phenotypic screening were collected on antibiotic-selective plates before screening. Screening for mutants with reduced nematicidal activity was performed as described in Kurz et al. (2003), and screening for mutants with reduced antibacterial activity was performed as described by Zhou et al. (2011). To confirm that mutant phenotypes were associated with single insertions and to ensure association between mutation and phenotype, the mutations were transduced into the parent genetic background using the generalized transducing bacteriophage ΦMAM1, as described in Matilla and Salmond (2014). Random-primed PCR and amplicon sequencing were used to identify transposon insertion points, as previously described Matilla et al. (2012). The genome of *S. plymuthica* A153 has been recently sequenced (Matilla and Salmond, unpublished results).

### PHENOTYPIC AND VIRULENCE ASSAYS

Antibacterial and antifungal assays against *Bacillus subtilis* JH642 and *Verticillium dahliae* 5368, respectively, were performed as described in Matilla et al. (2012), at 25°C unless stated otherwise. Assays for fungicidal activity against yeast were performed in the same manner as the antibacterial assays, but using overnight cultures *S. cerevisiae* and *S. pombe* grown in YPD. *C. elegans* virulence assays were performed as previously described Matilla et al. (2012), with the exception that synchronized worms were obtained by egg-lay, as described by Gems et al. (1998), with synchronized populations allowed to develop at 25°C to induce the *fer-15* sterility phenotype. Large-scale phenotypic screens for nematicidal activity were performed using around 50 synchronized L1-stage worms per well in 24-well plates (Kurz et al., 2003), where each well was inoculated with a transposon mutant for testing. Plates were incubated at 25°C and the survival of the worms was scored after 24 and 48 h. A Mantel-Cox log-rank test was used for statistical analysis of worm survival curves and was performed using Prism 5.0 (GraphPad Software). *P* values of 0.05 and below were considered statistically significant.

### MEASUREMENT OF β-GALACTOSIDASE ACTIVITY

Transcriptional fusion assays to detect expression of the *lacZ* reporter gene were performed as described previously (Ramsay, 2013), using the fluorogenic substrate 4-methylumbelliferyl β-D-galactoside (MUG2). Gene transcription values were expressed as relative fluorescence units (*r.f.u.)* s^-1^ OD_600_^-1^.

### QUANTIFICATION OF ZEAMINE ACTIVITY

Quantification of A153 supernatant bioactivity was performed using cultures of the andrimid-negative mutant, A153A. Bacterial cells were grow at 25°C, for 72 h, in MM supplemented with one of the 15 different carbon sources tested. Cultures were harvested after 72 h and culture supernatants were sterile-filtered (0.2 μm). Supernatant bioactivity (given as zeamine activity, Z_A_) was determined using a *B. subtilis* antibacterial bioassay (Matilla et al., 2012), and given as the fraction of the diameter of the resultant halo (H) and the diameter of the well (W) over the maximum OD_600_ attained by the bacteria in the relevant media.

### GENETIC COMPLEMENTATION OF Δhfq

For single-copy complementation of the in-frame *hfq* deletion mutant A153H, an inducible plasmid construct was first generated, using wild-type *hfq*. For this, the A153 *hfq* ORF was amplified using primers hfq-BamHI (5′-TAATTCCGCTAAGGGGCAATCTTTGCA-3′) and hfq-PstI (5′-TAATCTGCAGCTCGCAACGCGCTTTATTC-3′). The PCR product was digested with PstI and BamHI and inserted at the same sites in the vector pTRB30. The resulting plasmid, pJEEUH13, was introduced into A153H by electroporation. Gene expression was induced with 0.1 mM IPTG.

## RESULTS

### CHARACTERIZATION OF A153 FAST-KILLING OF *Caenorhabditis elegans*

**FIGURE 1:**
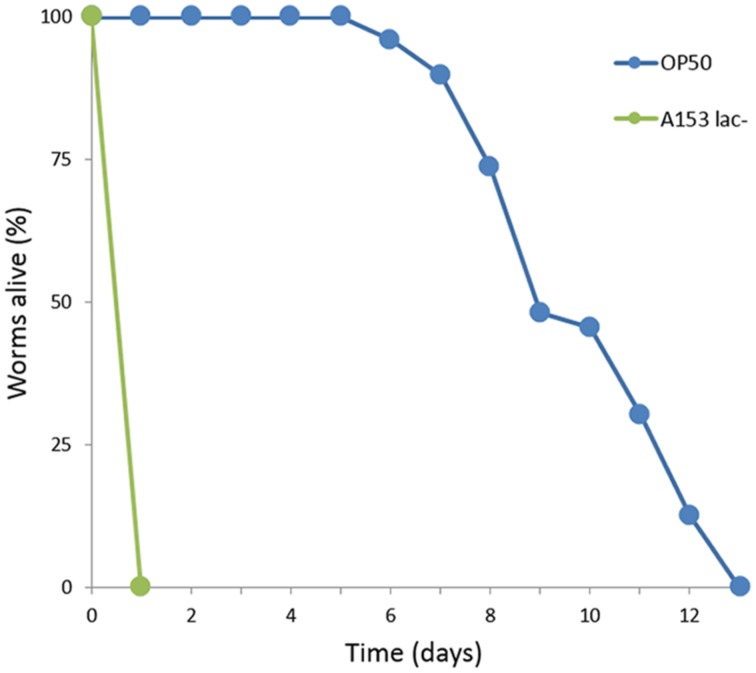
***Serratia plymuthica A153 antagonizes *Caenorhabditis elegans*.*** Survival of *C. elegans* when cultured on *S. plymuthica* A153. The results of a representative trial with at least 50 worms under each condition is shown.

It has been reported previously that *Serratia* spp. such as *S. marcescens* (Kurz and Ewbank, 2000) and *Serratia* sp. ATCC 39006 (Coulthurst et al., 2004) are capable of killing *C. elegans* over the span of 3–5 days by establishing an infection in the nematode intestine. We observed that *S. plymuthica* A153 is capable of killing worms quickly, within hours (**Figure 1**). To better understand this interaction, the A153 killing dynamics of *C. elegans* were subjected to an initial characterization, whereby *C. elegans* L4 larvae were transferred from the standard *C. elegans* food-source *E. coli* OP50 onto lawns of A153. After transfer, the worms succumbed in stages. Initially, they were strongly repulsed by the bacteria and showed strong avoidance of the bacterial lawn. The immediacy of this effect suggests that the bacteria may produce odorants or surfactants that deter nematode grazing (Pradel et al., 2007; Burlinson et al., 2013). Worms that remained on the bacterial lawn quickly become immobilized, within hours of transfer, and remained alive for a latency period of about 6 h, before they started dying. While immobilized, but before death, worms showed little to no spontaneous movement, although the pharynx and body wall muscles could be induced to contract by the gentle touch of an instrument. The majority of worms were dead within 24 h of transfer. The speed of A153 killing of *C. elegans* suggested that A153 produces a fast-acting nematicide that functions as a potent virulence factor in the nematode infection model.

### *Caenorhabditis elegans* SHOW AGE-DEPENDENT SENSITIVITY TO A153 FAST-KILLING

**FIGURE 2:**
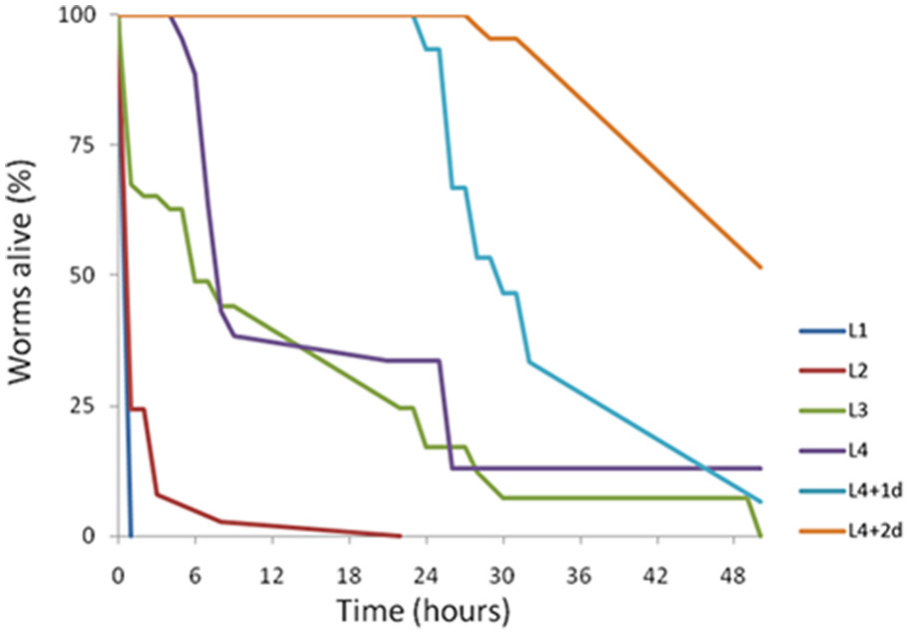
***Caenorhabditis elegans shows age-dependent sensitivity to the A153 nematicide**.* Survival of different larval stages and adult *C. elegans* worms when cultured on *S. plymu hica* A153. The results of a representative trial with at least 50 worms under each condition is shown.

The age of individual *C. elegans* has been shown previously to determine worm susceptibility to bacterial pathogens. Some fast-killing bacterial toxins such as pyocyanin have been shown to be more toxic to younger worms (Mahajan-Miklos et al., 1999). In contrast, under slow-killing infection models, older worms are generally more sensitive (Laws et al., 2004; Portal-Celhay et al., 2012). To determine if there were any age-dependent susceptibility effects to A153 fast-killing of *C. elegans*, worms from each of the four *C. elegans* larval stages (L1–L4) and day-1 and day-2 adults were transferred onto lawns of A153 and nematode survival was assayed hourly. The results revealed that *C. elegans* show significant age-dependent differential susceptibility to the A153 nematicide, with early larvae being more sensitive than late larvae (**Figure 2**; L1 vs. L4: *P* < < 0.05), and with larvae being more sensitive than adult worms (**Figure 2**; L4 vs. d1: *P* < < 0.05). This shows that A153 fast-killing and *C. elegans* susceptibility to the A153 nematicide is inversely correlated with the developmental stage and age of the worm.

### ISOLATION OF A153 MUTANTS WITH REDUCED VIRULENCE AGAINST *C. elegans*

**FIGURE 3:**
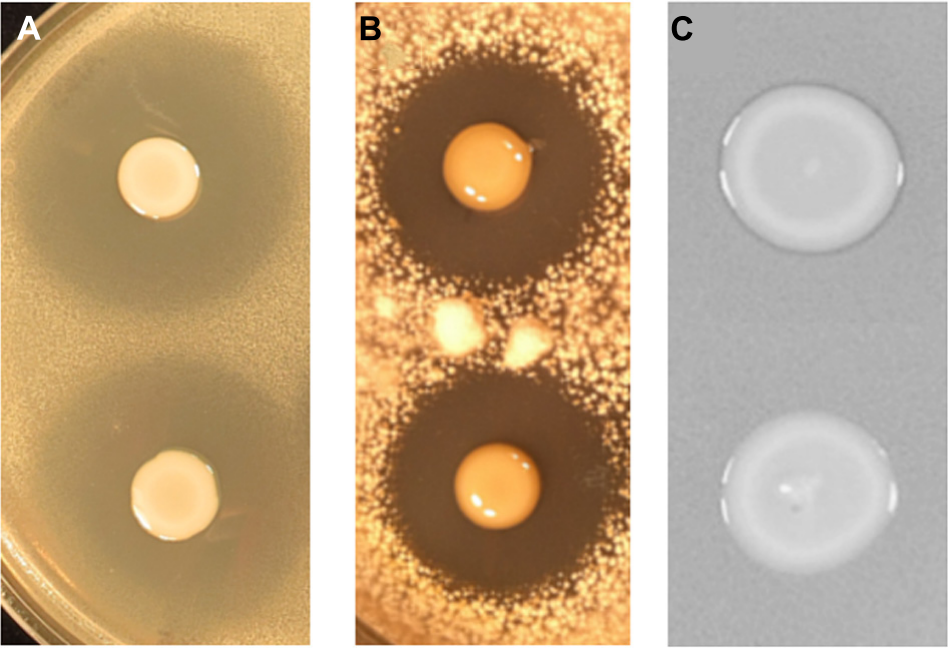
***Serratia plymuthica A153 mutants that show reduced virulence in *C. elegans* are specifically attenuated for zeamine production.*** Plate-based bioassays showing andrimid **(A)** oocydin A **(B)** and zeamine **(C)** production. **(A,B)** The upper culture spot shows the A153 wild-type whereas the lower culture spot shows the derived mutant A153Ce10. **(C)** The upper culture spot shows the A153 *ΔandR* mutant A153A whereas the lower culture spot shows the derived mutant A153Ce10A, which is representative of all mutants characterized in this study. In all cases, plates were incubated at 25°C either overnight **(A,C)** or for 5 days **(B)**.

To identify the genes involved in A153 fast-killing of *C. elegans*, a mutant library was generated using random transposon mutagenesis. In an initial screen, the library was screened looking for mutants with reduced nematicidal activity against L1-stage larvae. This approach yielded one mutant, A153Ce10, which showed significantly reduced ‘fast-killing’ of worms. This mutant still showed wild-type antibacterial (**Figure 3A**) and antifungal (**Figure 3B**) activities, suggesting that A153Ce10 is specifically deficient in worm-killing. Random-primed PCR confirmed that the transposon in A153Ce10 was in the gene *zmn16*, encoding a putative thioester reductase, and forming part of a hybrid FAS/PKS/NRPS gene cluster responsible for the biosynthesis of the broad-spectrum antibacterial antibiotic, zeamine (zmn), which was first described by Masschelein et al. (2013).

**FIGURE 4:**
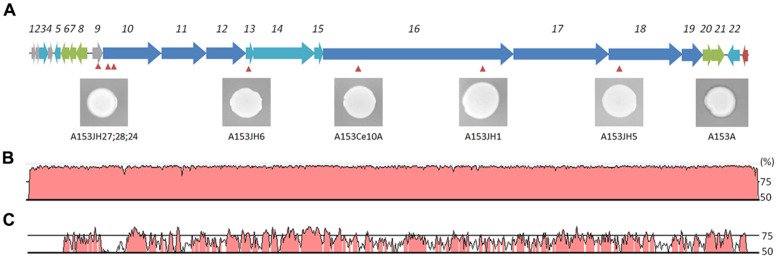
**The zeamine gene cluster is present in *Serratia* and *Dickeya* strains.** Genetic organization of the *zmn* gene cluster sequence in *S. plymuthica* A153 **(A)**. Location of the Tn-KRCPN1 transposon insertions are indicated by red arrowheads. Inserts show mutant antibacterial phenotypes against *B. subtilis* after an overnight incubation at 25°C. Note the panel depicting the phenotype of the A153 *ΔandR* parent strain to the far right. DNA homology (%) between the *zmn* gene cluster of A153 and those of *S. plymuthica* RVH1 **(B)** and *D. solani* MK10 **(C)** is presented. Sequence comparisons were performed using wgVISTA and show regions of >50% homology.

**FIGURE 5:**
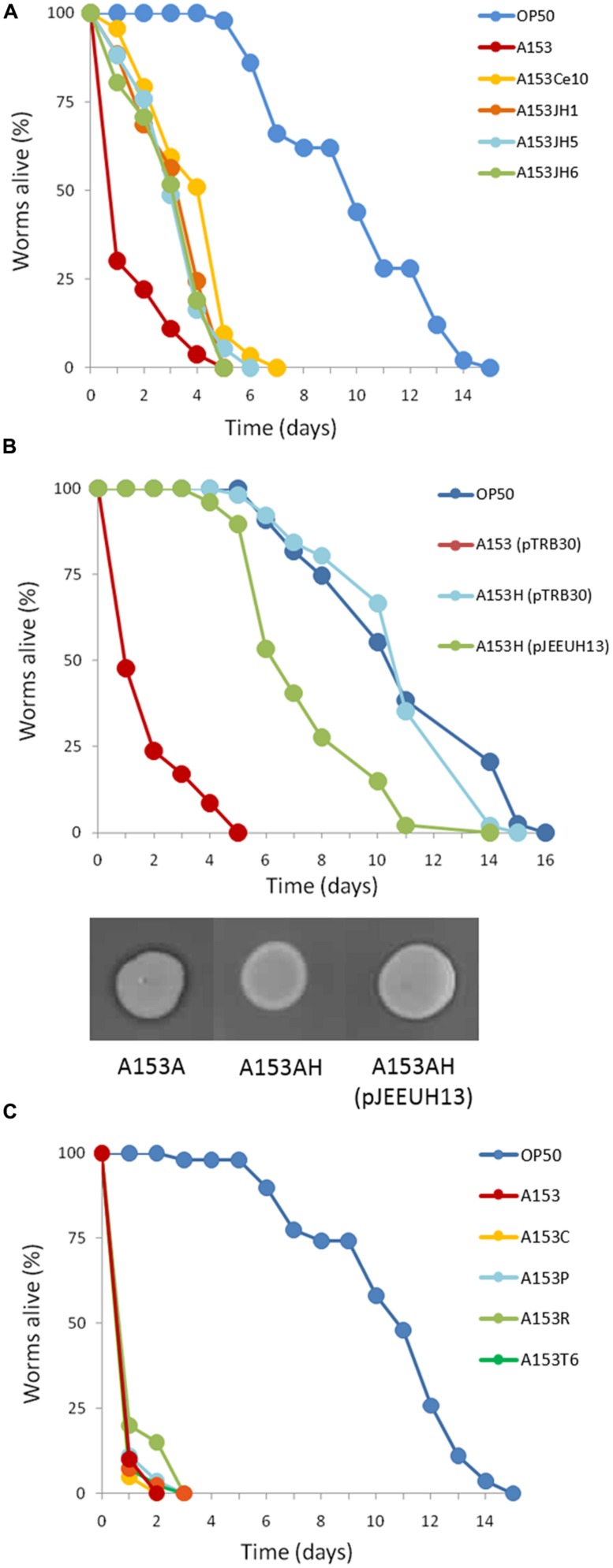
**Kinetics of the nematicidal properties of *Serratia plymuthica* A153 strains. (A)** Mutation of the *zmn* gene cluster attenuates *S. plymuthica* A153 fast killing of *C. elegans*. Compared with the A153A parent strain, mutants A153JH1, A153JH5, A153JH6 and A153Ce10A, show reduced virulence in *C. elegans* (*P* < < 0.05).** (B)** Mutation of the chaperone Hfq eliminates A153 virulence in *C. elegans*. The virulence could be partially restored by the *in trans* expression of *hfq* (using plasmid pJEEUH13). Gene expression was induced using 0.1 mM IPTG. Inserts show antibacterial phenotypes of the parent and complemented strains after an overnight incubation at 25°C. **(C)** Mutation of the regulators *rpoS*, *pigP,* and *csrB* had no effect on A153 fast-killing of *C. elegans,* and neither did deletion of the A153 T6SS. For each experiment, worms were transferred at the L4 stage and the results of one representative trial with at least 50 worms under each condition is shown.

Zeamine was first identified in the plant pathogen *Dickeya zeae* EC1 as a phytotoxic virulence factor with antibacterial properties (Wu et al., 2010; Zhou et al., 2011), and our results suggest that zeamine also has nematicidal activity. In A153, the antibacterial activity of zeamine was masked by the production of another potent antibiotic, andrimid (cf. **Figures 3A,C**; Matilla and Salmond, unpublished results). Using a mutant defective in the production of this antibacterial compound, A153A, a random transposon mutant library was constructed and screened for mutants lacking the small halo associated with the production of zeamine by A153 (**Figure 3C**). Six mutants showing no, or reduced, antibacterial activity (A153JH1, A135JH5, A153JH6, A153JH24, A153JH27, and A153JH28) and with transposon insertions in the *zmn* gene cluster (**Figure 4A**) were isolated. These mutants showed significantly reduced nematicidal activity compared to that of the A153A parent strain (*P* < < 0.05; **Figure 5A**, Figure S1), confirming that zeamine is indeed the A153 nematicide and responsible of the observed A153 fast-killing of *C. elegans*.

The A153 *zmn* gene cluster spans over 54 kb and contains 22 genes (*zmn1-22*), organized into three putative operons (**Figure 4A**; Table S1). As previously described in *S. plymuthica* RVH1, the A153 *zmn* gene cluster contains the genes for three multidomain PKSs (*zmn10, zmn11, zmn18*) and two multidomain NRPSs (*zmn16, zmn17*). In addition to these biosynthetic genes, the cluster also contains genes encoding modifying enzymes (*zmn3, zmn12, zmn14, zmn15, zmn22*) and transport-related proteins (*zmn7, zmn8, zmn9, zmn20, zmn21*) – the latter with a proposed role in conferring innate resistance to the zeamine antibiotic (Masschelein et al., 2013). A putative integrase-encoding gene marks the downstream end of the cluster, suggesting that it could have been acquired by horizontal gene transfer.

### THE *zmn* GENE CLUSTER IS WIDELY DISPERSED WITHIN *Serratia* AND *Dickeya* GENERA

Genome comparison analyses revealed that the *zmn* gene cluster is present in *S. plymuthica* strains AS9, AS12, AS13, A30, S13, and V4 (Table S2). Additionally, we also identified the biosynthetic gene cluster in several phytopathogenic strains belonging to the *Dickeya* genus, including *D. solani* MK10, MK16, IPO222; *D. zeae* DZ2Q and ZJU1202; and *Dickeya* spp. MK7 and NCPPB 3274 (Table S2). The *Serratia* and *Dickeya zmn* clusters span between 50.64- and 54.02-Kbp and are between 59.7 and 96.4% identical at the DNA level with the A153 *zmn* gene cluster (Table S3).

**FIGURE 6:**
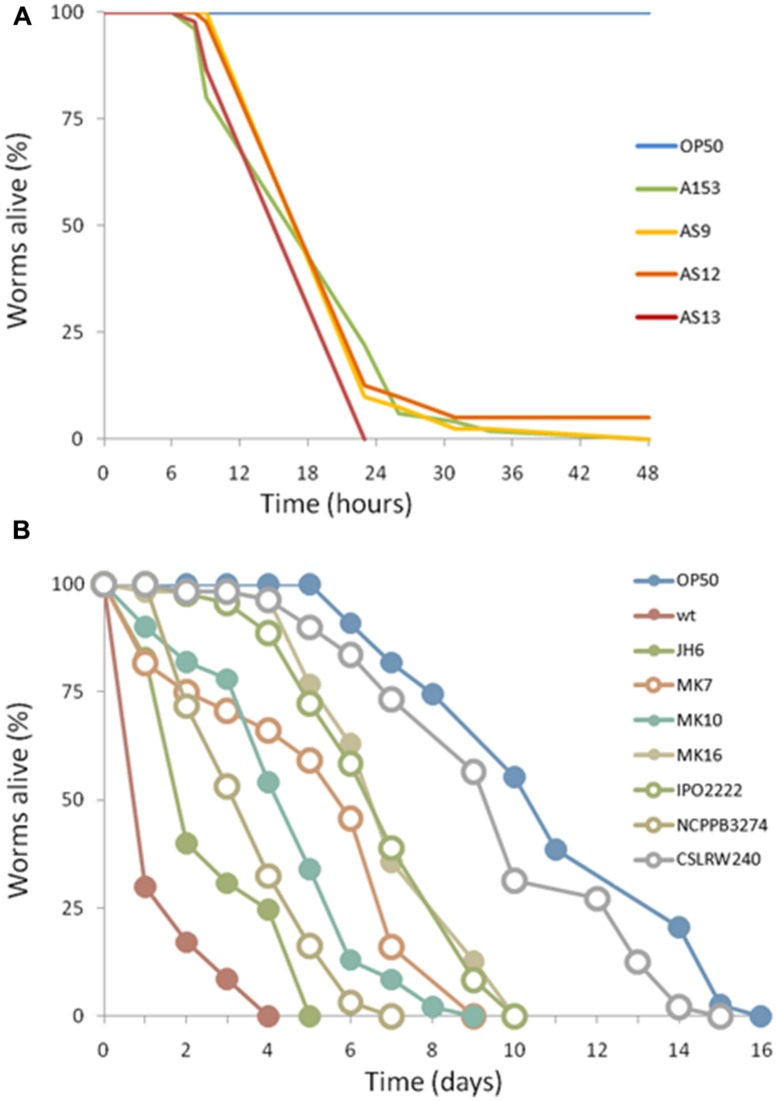
**Virulence of enterobacterial strains harboring the *zmn* gene cluster.** The pigmented *S. plymuthica* strains AS9, AS12 and AS13 show fast killing of *C. elegans* **(A)**. In contrast, *D. solani* strains MK10, MK16, IPO2222 and *Dickeya* spp. MK7 and NCPPB3274 showed no fast killing of *C. elegans* **(B)**. For each experiment, worms were transferred at the L4 stage and the results of a representative trial with at least 50 worms under each condition is shown.

*In silico* analyses showed that the A153 and RVH1 *zmn* gene clusters have the same gene and domain organization (**Figure 4B**). However, we found that the A153 PKS Zmn10 contains a previously unidentified dehydratase (DH) domain. Based on the high sequence identity (around 95%) between *zmn* gene clusters in A153 and related *S. plymuthica* strains, we hypothesized that all these strains could possess high nematicidal activities. Thus, using L4-stage larvae, we showed that *S. plymuthica* strains AS9, AS12, and AS13 are highly virulent and capable of killing *C. elegans* at similar levels to those of A153, with 50% of worms dying within 18 h of transfer (**Figure 6A**).

Importantly, pairwise comparisons *in silico* indicate that genes *zmn1-4* are not present in strains of *Dickeya* and that the *zmn5* homolog constitutes the first gene of these *zmn* gene clusters (**Figure 4C**). In addition, whereas the *zmn* gene clusters in different strains of *Dickeya* show around 60% sequence identity with the A153 cluster, the putative permease Zmn9 is only about 50% conserved between the genera. The NCBI conserved domains database predicts that the *Serratia* Zmn9 contains a zinc-dependent phospholipase domain, whereas Zmn9 in *Dickeya* is predicted to contain a CDP-alcohol phosphatidyltransferase domain. To investigate if strains of *Dickeya* show similar nematicidal differences to *zmn*-carrying strains of *Serratia* despite these differences, a subset of *Dickeya* strains that carry the *zmn* gene cluster (MK7, MK10, MK16, IPO2222, and NCPPB 3274) were chosen for *C. elegans* virulence assays. Interestingly, although the tested *Dickeya* strains had variable pathogenic capacity against L4-stage *C. elegans* (**Figure 6B**), none of them was found to show the fast-killing phenotype observed in strains of *S. plymuthica* that carry the *zmn* gene cluster.

### THE *zmn* GENE CLUSTER IS TRANSCRIBED IN A GROWTH PHASE-DEPENDENT MANNER

**FIGURE 7:**
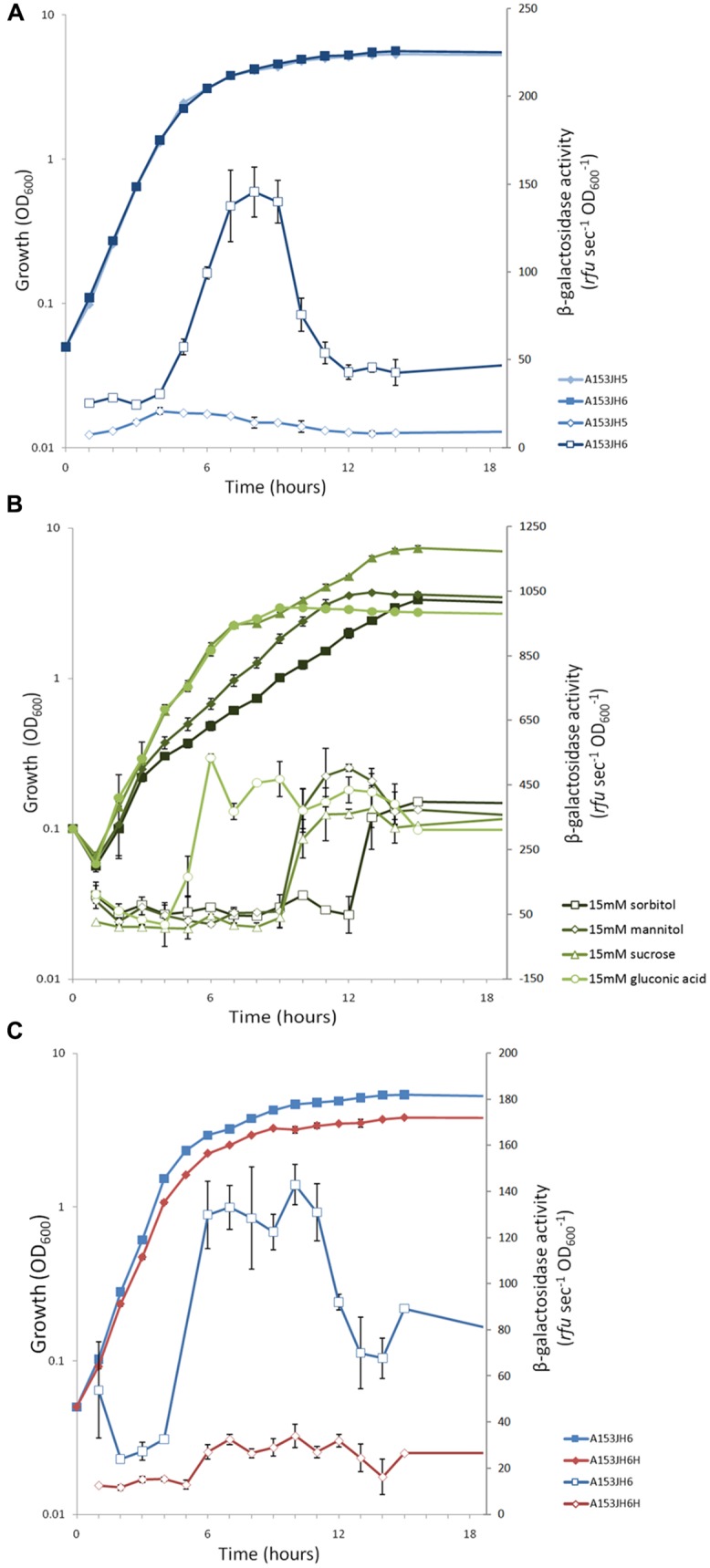
**The expression of the *S. plymuthica* A153 *zmn* gene cluster is growth phase-dependent and post-transcriptionally regulated by the RNA chaperone Hfq. (A)** Transcription of the *zmn* gene cluster throughout growth in *Serratia plymuthica* A153. The dip in β-galactosidase activity post-peak suggests the enzyme is subject to proteolytic turnover, which has been observed previously in A153 (Matilla and Salmond, unpublished results). **(B)** Different carbon sources have different effects on the correlation between *zmn* gene transcription and the bioactivity of A153A-derived supernatants. β-galactosidase activity was measured in strain A153JH6 grown in 1C-OMM supplemented with 15 mM sorbitol, mannitol, sucrose, or gluconic acid. **(C)** Expression of the *zmn* gene cluster is regulated by the RNA chaperone Hfq. β-galactosidase activity was measured in strain A153JH6 in the presence (red) or absence (blue) of a chromosomal *hfq* gene deletion. **(A,B)** The strains were grown in LB at 25°C and β-galactosidase activities were measured in strains expressing chromosomal *zmn13::lacZ* fusions. In all panels solid symbols represent growth of the corresponding strains whereas open symbols represent β-galactosidase activity. Data shown are the average values ±SD of at least three experiments.

To investigate the transcription of the *zmn* gene cluster, β-galactosidase activity was measured in a chromosomal *zmn13*::*lacZ* fusion strain (strain A153JH6). Transcription of *zmn* biosynthetic genes started in mid-logarithmic phase of growth and reached an apparent maximum in early stationary phase of growth (**Figure 7A**). The sharp decrease in β-galactosidase levels beyond this point may be explained by proteolytic turnover of β-galactosidase (Matilla and Salmond, unpublished results).

### THE PRODUCTION OF ZEAMINE IS CARBON SOURCE-DEPENDENT

Zeamine production has been shown previously to differ between growth media, being higher in *D. zeae* EC1 when grown in an OMM, compared to the standard LB culture medium (Zhou et al., 2011). Thus, we investigated zeamine production in strains grown in different carbon sources by determining the bioactivity of cell-free supernatants against *B. subtilis,* which is sensitive to zeamine (**Figure 3C**). Our results showed that the production of the bioactive molecule is carbon source-dependent, with some carbon sources favoring high levels of zeamine biosynthesis (e.g., sorbitol and mannitol) whereas others do not support the production of zeamine at all (e.g., gluconic acid; Table S4). Expression of the *zmn* gene cluster, reported using β-galactosidase assays, was examined in a modified OMM with different carbon sources (1C-OMM). Unexpectedly, no correlation between *zmn* gene transcription and zeamine production was observed (**Figure 7B**; Table S4).

### THE RNA CHAPERONE Hfq REGULATES THE PRODUCTION OF ZEAMINE AND THE EXPRESSION OF *zmn* BIOSYNTHETIC GENES

The RNA chaperone Hfq acts as a regulator of gene expression by interacting with small regulatory RNAs to stabilize the interaction between these and their target mRNAs through the formation of regulatory RNA-RNA complexes (Vogel and Luisi, 2011). Mutants defective in *hfq* are highly pleiotropic and can be attenuated in both virulence and the production of secondary metabolites in *Serratia* sp. ATCC39006 (Wilf et al., 2012). To investigate whether Hfq is involved in regulating the production of zeamine in *S. plymuthica* A153, *C. elegans* virulence assays were performed. The results showed that deletion of *hfq* in strain A153H strongly attenuated virulence to *C. elegans* L4 larvae (*P* < < 0.05; **Figure 5B**). The virulence of A153H could be partially restored by expressing *hfq in trans* (**Figure 5B**). β-galactosidase assays showed that deletion of *hfq* abolished the transcription of the *zmn* gene cluster (**Figure 7C**), confirming that Hfq positively regulates the production of zeamine. It is well known that Hfq regulates the translation of the stationary-phase sigma factor RpoS (Vogel and Luisi, 2011). To investigate whether Hfq regulation is dependent on RpoS, we phenotypically characterized an *rpoS* mutant in A153. However, this mutant showed the same antibacterial and nematicidal activities as the wild-type strain (**Figure 5C**). Mutants defective in the non-coding small RNA *csrB* (Babitzke and Romeo, 2007) and the transcriptional regulator PigP (Fineran et al., 2005) were also unaffected in their virulence against *C. elegans* (**Figure 5C**).

### ZEAMINE IS TOXIC TO ASCOMYCETE YEASTS

**FIGURE 8:**
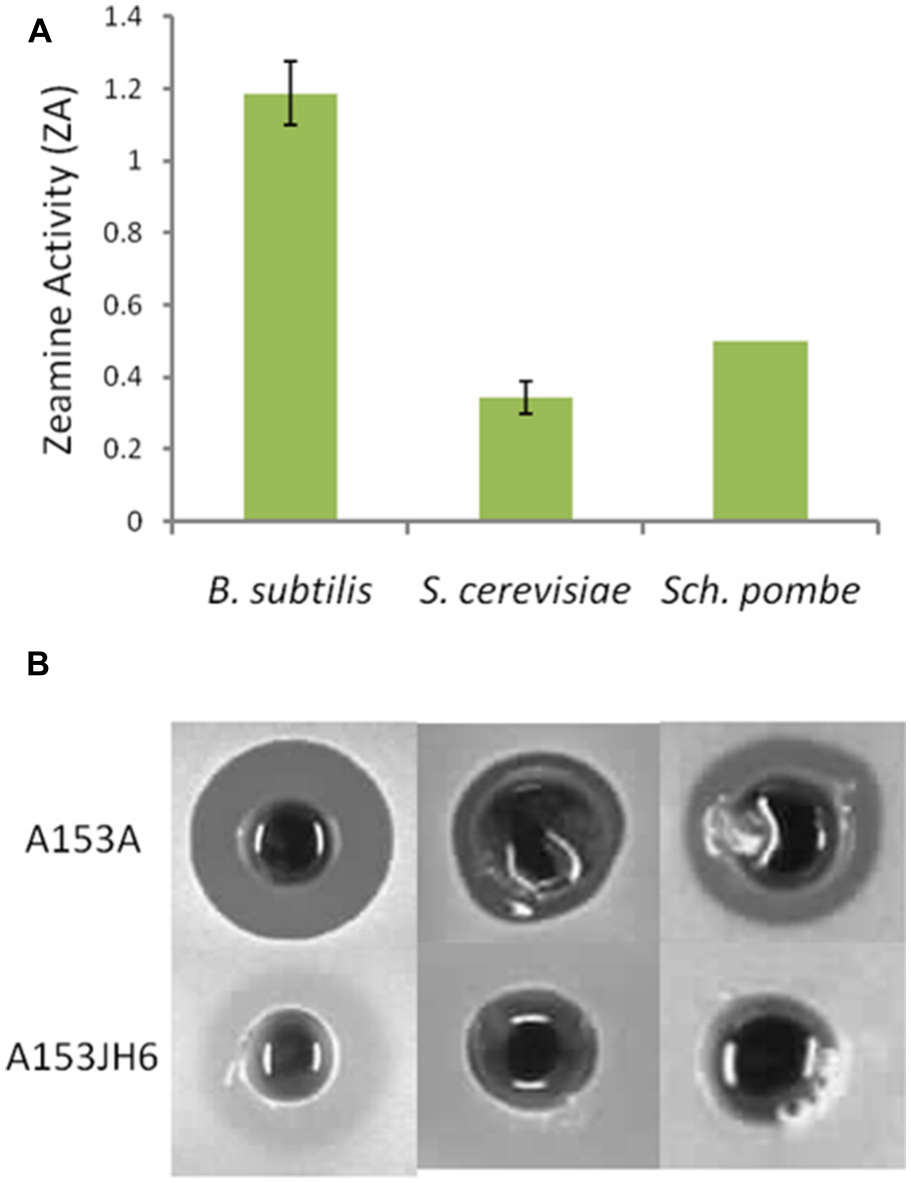
**Zeamine is toxic to the ascomycete yeasts *Saccharomyces cerevisiae* and *Schizosaccharomyces pombe*. (A)** Bioactivity of cell-free supernatants of A153A show bioactivity against *Bacillus subtilis* and the ascomycete yeasts *S. cerevisiae* and *S. pombe*. A153 strains were grown in OMM at 25°C for 48 h and the bioactivities were quantified from bioassay plates incubated overnight at 30°C. Data shown are average values ±SD from at least three experiments. **(B)** Supernatants from stationary phase cultures of A153JH6 grown under the same conditions produce no antibiotic halos on bioassay plates, confirming that the halos are produced by zeamine.

Zeamine shows some structural similarity to another family of hybrid polyamino-polyketides: fabclavines. These natural products have been shown to have broad-spectrum antibiotic activity against a diverse set of organisms including bacteria and ascomycete fungi (Fuchs et al., 2014). Using cell-free supernatants of A153A and A153JH6, we showed that zeamine is bioactive against the ascomycete yeasts *S. cerevisiae* and *S. pombe* (**Figure 8**).

### THE TYPE VI SECRETION SYSTEM OF *S. plymuthica* A153 IS NOT INVOLVED IN VIRULENCE

The bacterial Type VI secretion system (T6SS) is the most recently described secretion system in Gram-negative bacteria and has been found to promote bacterial virulence against both prokaryotic competitors and eukaryote hosts (Coulthurst, 2013). *In silico* analyses revealed that a T6SS gene cluster highly homologous to that present in *Citrobacter rodentium* ICC168 is present in the genome of A153 (Matilla and Salmond, unpublished results). The role of the T6SS in virulence against nematodes remains largely uncharacterized but Sana et al. (2012) showed that it plays a role in *P. aeruginosa* “slow killing” of *C. elegans*. To investigate if the *S. plymuthica* A153 T6SS is involved in virulence, an A153 T6SS mutant was constructed (A153T6) and characterized. However, no difference in the virulence was observed between A153T6 and the A153 wild-type (**Figure 5C**).

## DISCUSSION

Although zeamine was first described as a phytotoxin with broad-spectrum antibacterial properties (Wu et al., 2010; Zhou et al., 2011), this study showed that zeamine is also a potent nematicide. Furthermore, it is a characteristic of some bacterial toxins that younger hosts are more susceptible than older ones (Mahajan-Miklos et al., 1999), and consistent with this, *C. elegans* showed age-dependent sensitivity to the A153 nematicide. In addition, we have shown that zeamine is capable of killing *S. cerevisiae* and *S. pombe*, and therefore zeamine, by definition, is also a fungicide. This leads us to conclude that zeamine is a very broad-spectrum antibiotic that is capable of antagonizing a phylogenetically diverse set of organisms, making it unsuitable, in an unmodified form, for application as a therapeutic antibiotic.

Like the fabclavines (Fuchs et al., 2014), the zeamine molecule has a polyaminated fatty acid backbone that is derived from modified secondary lipid metabolism (Wu et al., 2010; Masschelein et al., 2013). Zeamine also shows some structural similarity with compounds such as the phytotoxin syringomycin, which has been shown to antagonize plant cells by forming ion channels in the plant cell membrane (Hutchison and Gross, 1997). Altogether, it is possible that zeamine has lipophilic properties and might interact with the lipids of cell membranes through a mechanism analogous to that of cationic antimicrobial peptides (Hancock, 2001). A membranal target would be entirely consistent with the observed strong susceptibility of very diverse organisms to zeamine.

Fatty acids have been shown previously to function as nematicides against various phytopathogenic nematodes, and have been hypothesized to disrupt plasma membranes to facilitate solubilization of the nematode cuticle or hypodermis (Anke et al., 1995; Davis et al., 1997). Similarly, cationic peptides are capable of interacting with and disrupting cell membranes owing to a three-dimensional amphiphilic structure (Joondan et al., 2014), and various classes thereof have been shown effective against fungi, protozoa and mammalian cells (Hancock, 2001). Intriguingly, cationic peptides share many of these properties with zeamine, and have been shown previously to function as nematicides (Colgrave et al., 2008), and are capable of causing severe damage to the intestine of lepidopteran larvae (Barbeta et al., 2008). It is therefore possible that the nematicidal cytotoxicity of zeamine involves damage to, and vacuolisation of, the cells that line the *C. elegans* intestine.

Our results raise questions about the role of zeamine in nature. The majority of sequenced *S. plymuthica* strains that carry the *zmn* gene cluster were isolated from agricultural contexts, such as the plant antho- and rhizosphere (e.g., Åström and Gerhardson, 1988; Fürnkranz et al., 2012; Neupane et al., 2012a,b,c). Plant root exudates are rich in sugars and other organic nutrients that favor root colonization by soil-borne bacteria (Bais et al., 2006), and this study has shown that different sugars have different effects on zeamine production by A153 – with some repressing the production of the antibiotic whilst others favor it. In this regard, it is notable that A153 was initially isolated on the basis that it antagonized plant growth (Åström and Gerhardson, 1988), and that zeamine has been found to be a potent phytotoxin, capable of antagonizing both shoot and root development in rice seedlings (Zhou et al., 2011). Together with our results showing that zeamine is also a potent nematicide, these observations raise the question: are there any possible large-scale effects of zeamine production by rhizosphere-associated soil bacteria?

Various *Dickeya* spp. are pathogens of plants, and as some of these have been found to contain the *zmn* gene cluster, their nematicidal properties were investigated. However, contrary to expectation, none of the selected strains were found to show fast-killing of *C. elegans*. The absence of genes *zmn1-4* in strains of *Dickeya* suggests that the cryptic *zmn* gene clusters of the assayed *Dickeya* isolates may not be effectively or functionally expressed, correlating with a lack of rapid nematicidal activity. Alternatively, and considering the low sequence conservation between *Serratia* and *Dickeya zmn9*, it is possible that the final biosynthetic products of the *zmn* gene clusters of the *Dickeya* strains analyzed in this study do not possess the same biological properties as zeamine. For example, Masschelein et al. (2013) showed that *S. plymuthica* RVH1 is capable of producing three different zeamine molecules (zeamine, zeamine I, and zeamine II), of which zeamine I is the predominant molecule produced by *D. zeae* EC1 (Wu et al., 2010). The individual contributions of these molecules to the antibiotic activity of zeamine, *sensu lato*, is unclear, although further work on this topic might reveal differential toxic activities between different zeamines and derived molecules. If different zeamine species are found to show differential specific toxicities, this would raise the possibility that these may be used as more-specific antibiotics or pesticides. It is also possible that the *zmn* gene clusters of the *Dickeya* isolates tested in this study are simply cryptic under the conditions tested – as is known for gene clusters encoding other secondary metabolites (Osbourn, 2010). It is, however, clear that various *Dickeya* spp. are virulent in the *C. elegans* model, consistent with previous work that found that the plant pathogens *D. dadantii* 3937c, *Agrobacterium tumefaciens* CFBP2413, and *Pectobacterium carotovorum* CFBP 2141, are capable of killing *C. elegans* through infection (Couillault and Ewbank, 2002).

The expression pattern of the A153 *zmn* gene cluster is characteristic for secondary metabolite antibiotics, which are typically produced during conditions of nutrient limitation and reduced growth (Bibb, 2005). As is common with secondary metabolites, transcription of *zmn* biosynthetic genes is sensitive to environmental conditions. In contrast to findings in other bacteria such as *S. plymuthica* RVH1 (Masschelein et al., 2013), *zmn* gene transcription in A153 does not appear to be thermoregulated (Figure S2). Zeamine production does, however, show media-dependent effects in A153, as has previously been reported for *D. zeae* EC1 (Zhou et al., 2011). The basis of this effect appears to be post-transcriptional, as we did not observe a correlation between *zmn* gene transcription and zeamine bioactivity between different carbon sources (cf. **Figure 7B**; Table S4).

We observed a correlation between *zmn* gene transcription and the activity of the RNA chaperone Hfq – with the A153 *zmn* gene cluster being silent in a *Δhfq* background – consistent with its role as a regulator of secondary metabolism in other species of *Serratia.* Mutation of *hfq* has been shown previously to reduce the transcription of genes involved in iron uptake in *E. coli* (Večerek et al., 2003), and intriguingly, as part of our mutagenesis program, we isolated a mutant (A153JH11) with a transposon insertion in the A153 homolog of the ferric aerobactin receptor (Figure S3A), which showed increased production of zeamine (Figure S3B), suggesting that production of the antibiotic is increased during conditions that might mimic iron limitation.

In summary, the work presented in this study has shown that *S. plymuthica* A153 produces the very broad-spectrum antibiotic, zeamine. Although zeamine was initially described as a phytotoxin with antibacterial activity, our results show that zeamine is also a potent nematicidal compound and antifungal. That zeamine antagonizes such a phylogenetically diverse set of organisms suggests that it targets a highly conserved cellular process, which would make it unsuitable as a specific antibiotic. That target is likely to be the cell membranes of diverse hosts. However, the research presented here may help toward the development of zeamine analogs with enhanced host specificity in nematodes and fungi.

## Conflict of Interest Statement

The authors declare that the research was conducted in the absence of any commercial or financial relationships that could be construed as a potential conflict of interest.
