# Supplementary material for: The broad-spectrum antibiotic, zeamine, kills the nematode worm Caenorhabditis elegans
Source: Front Microbiol. 2015 Feb 26;6:137. doi: 10.3389/fmicb.2015.00137 (PMC4341552; doi:10.3389/fmicb.2015.00137)
Supplement: Supplementary file 1 [file presentation_1.pdf]

Supplementary Material

The broad-spectrum antibiotic, zeamine, kills the nematode worm *Caenorhabditis elegans*

Josephine E. E. U. Hellberg[1], Miguel A. Matilla[1,2]*, George P. C. Salmond[1]*

[1]Department of Biochemistry, University of Cambridge, Cambridge, UK.
[2]Department of Environmental Protection, Estación Experimental del Zaidín – Consejo Superior de Investigaciones Científicas, Granada, Spain.

*** Correspondence:**
Professor George P. C. Salmond, Department of Biochemistry, University of Cambridge, Tennis Court Road, Cambridge CB2 1QW, UK.
e-mail: gpcs2@cam.ac.uk;
Dr. Miguel A. Matilla, Department of Environmental Protection, Estación Experimental del Zaidín – Consejo Superior de Investigaciones Científicas, Profesor Albareda 1, Granada 18008, Spain.
e-mail: miguel.matilla@eez.csic.es

1. Supplementary Figures and Tables

1.1. Supplementary tables

Table S1. Predicted functions of open reading frames in the *S. plymuthica* A153 *zmn* biosynthetic gene cluster.

Protein	Size (aa)	Proposed function (domain(s))[a]	Sequence similarity (protein, *origin*)	Identity/ Similarity (%)	GenBank™ accession no.
Zmn1	141	Hypothetical protein			
Zmn2	170	Hypothetical protein			
Zmn3	457	Pyridoxal-dependent decarboxylase	WP_014808925, *Desulfomonile tiedjei*	97/98	WP_014808925
Zmn4	217	Putative N-acyltransferase	WP_011612911, *Trichodesmium erythraeum*	25/45	WP_011612911
Zmn5	242	4'-phosphopantetheinyl transferase	EntD, *Enterobacter sp.* DC3	48/63	AZXZ01000033.1
Zmn6	306	HlyD family secretion protein	HlyD, *Asticcacaulis sp*. AC406	37/58	WP_031237376
Zmn7	239	ABC transporter, ATPase component	SalX, *Dickeya zeae*	69/78	WP_016943529
Zmn8	367	ABC transporter membrane protein	DevC, *Dickeya solani*	71/83	WP_022632847
Zmn9	331	Putative permease	CDL85995, *Xenorhabdus cabanillasii* JM26	42/61	CDL85995
Zmn10	2264	PKS (KS-AT-ACP-KR-DH)	PfaA, *Photorhabdus temperata* subsp. *khanii* NC19	66/75	ETS29777
Zmn11	1436	PKS (KS-KS-DH)	WP_012777102, *Photorhabdus asymbiotica*	69/81	WP_012777102
Zmn12	1017	PfaD-family protein (NMO-AMT)	KER04067, *Photorhabdus temperata* subsp. *temperata* Meg1	79/86	KER04067
Zmn13	255	3-oxoacyl-ACP reductase (KR)	WP_012989343, *Xenorhabdus bovienii*	74/85	WP_012989343
Zmn14	412	Thioester reductase (TR)	HetM, *Dickeya solani*	67/80	WP_022632853
Zmn15	259	Carbon-nitrogen hydrolase	CDH00688, *Xenorhabdus bovienii* str. *feltiae* Moldova	66/80	CDH00688
Zmn16	4169	NRPS (A-T-C-A-T-C-A-T-C-C-A-T)	CDL85988, *Xenorhabdus cabanillasii* JM26	53/66	CDL85988
Zmn17	2180	NRPS (C-A-T-C-A-T)	CDL85987, *Xenorhabdus cabanillasii* JM26	61/72	CDL85987
Zmn18	1531	PKS (KS-AT-KR-ACP)	CDL85171, *Xenorhabdus szentirmaii* DSM 16338	57/69	CDL85171
Zmn19	512	Condensation domain-containing protein	CDL85985, *Xenorhabdus cabanillasii* JM26	50/66	CDL85985
Zmn20	314	ABC transporter, ATP-binding cassette	WP_022632859, *Dickeya solani*	72/87	WP_022632859
Zmn21	371	ABC transporter, permease component	CDH18908, *Xenorhabdus bovienii* str. *kraussei* Quebec	70/84	CDH18908
Zmn22	365	Hydrolase family protein	WP_022632861, *Dickeya solani*	56/72	WP_022632861

[a] The following abbreviations are used: A, adenylation domain; ABC, ATP-binding cassette; ACP, acyl carrier protein domain; AMT, aminotransferase domain; AT, acyl transferase domain; C, condensation domain; DH, dehydratase domain; KR, ketoreductase domain; KS, ketosynthase domain; NMO, nitropropane monooxygenase domain; T, **thiolation** domain; TR, thioester reductase domain.

Table S2. Table of bacterial species and strains that contain the *zmn* gene cluster.

Strain	Origin (country)	Reference
Serratia plymuthica		
A153	Isolated from rhizosphere of winter wheat (Sweden)	Åström & Gerhardson (1988)
A30	Isolated from rotting potato tissue (Netherlands)	Czajowski et al. (2012)
AS9	Isolated from rapeseed roots (Sweden)	Neupane *et al.* (2012a)
AS12	Isolated from rapeseed roots (Sweden)	Neupane *et al.* (2012b)
AS13	Isolated from rapeseed roots (Sweden)	Neupane *et al.* (2012c)
RVH1	Isolated from a raw vegetable processing line (not specified)	Van Houdt *et al.* (2004)
S13	Isolated from oil pumpkin anthosphere (Austria)	Fürnkranz *et al.* (2012)
V4	Isolated from a milk processing plant (Portugal)	Cleto *et al.* (2012)
***Dickeya* spp.**		
MK7	Isolated from river water (United Kingdom)	Pritchard *et al.* (2013b)
NCPPB 3274	Isolated from Chinese evergreen (*Aglanoema*) (St. Lucia)	Pritchard *et al.* (2013b)
Dickeya solani		
GBBC 2040	Not specified	Pritchard *et al.* (2013a)
MK10	Isolated from potato (Israel)	Pritchard *et al.* (2013a)
MK16	Isolated from river water (United Kingdom)	Pritchard *et al.* (2013a)
IPO 2222	Isolated from potato (Netherlands)	Pritchard *et al.* (2013a)
Dickeya zeae		
EC1	Isolated from rice (China)	Hussain *et al*. (2008)
DZ2Q	Isolated from rice (Italy)	Bertani *et al*. (2013)
ZJU1202	Isolated from diseased rice (China)	Li *et al*. (2012)

Table S3. Comparison of *zmn* gene clusters in strains of *S. plymuthica* and *Dickeya* spp.

Strain	Length of cluster (kb)	Sequence identity with A153 (%)	Sequence identity with MK10 (%)
Serratia plymuthica			
A153	54.02	-	61.2
AS9	53.50	95.1	61.6
AS12	53.61	95.3	60.2
AS13	53.61	95.3	60.2
RVH1	53.85	96.4	61.0
S13	53.92	96.3	58.2
V4	53.68	95.9	61.5
***Dickeya* spp.**			
MK7	51.01	60.4	100.0
NCPPB 3274	51.06	60.2	96.6
Dickeya solani			
GBBC 2040	51.25	60.3	92.5
MK10	50.64	59.7	-
MK16	50.65	59.8	92.0
IPO 2222	51.26	60.3	92.5

Table S4. Zeamine activity (Z_A) of culture supernatants from strain A153A, grown in minimal medium supplemented with different carbon sources (15mM).

Carbon source	Z_A OD_{600}^{-1} [a]	Z_A category[b]
Arabinose	0.14	High
Citrate	0.00	None
Fructose	0.16	High
Galactose	0.02	Low
Gluconic acid	0.00	None
Glucose	0.03	Low
Glycerol	0.00	None
Lactose	0.02	Low
Maltose	0.08	Medium
Mannitol	0.11	Medium
Mannose	0.08	Medium
Sorbitol	0.14	High
Succinic acid	0.19	High
Sucrose	0.01	Low
Xylose	0.07	Medium

[a] To calculate this metric, the Z_A of culture supernatants grown for 72 hours was divided with the maximum OD_{600} observed in medium supplemented with the relevant carbon source.

[b] Z_A categories were delineated as follows: none (Z_A: 0.00); low (Z_A: 0.01-0.05); medium (Z_A: 0.05-0.12); high (ZA: 0.12 and above)

1.2. Supplementary figures

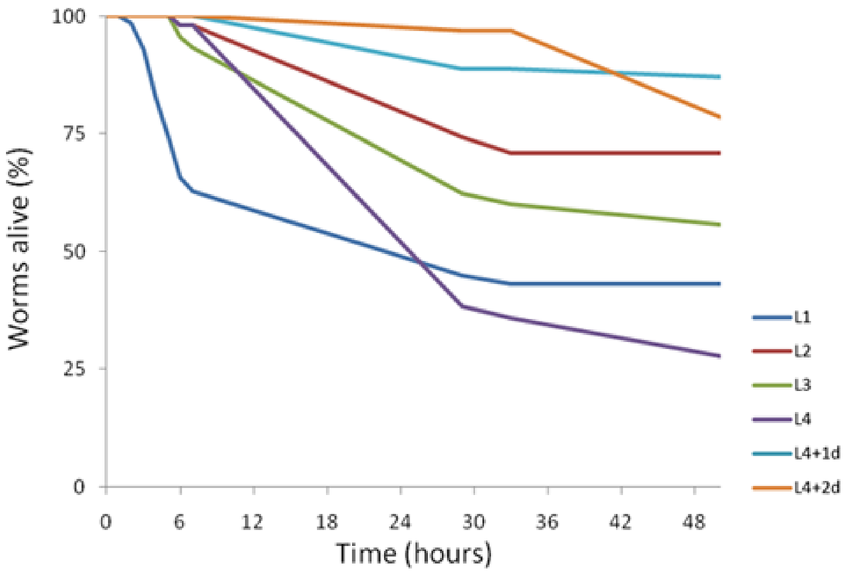

Figure S1. Mutation of the *zmn* gene cluster attenuates *C. elegans* age-dependent sensitivity to the A153 nematicide. Survival of different larval stages and adult *Caenorhabditis elegans* worms when cultured on *S. plymuthica* A153JH6. The results of a representative trial with at least 50 worms under each condition is shown.

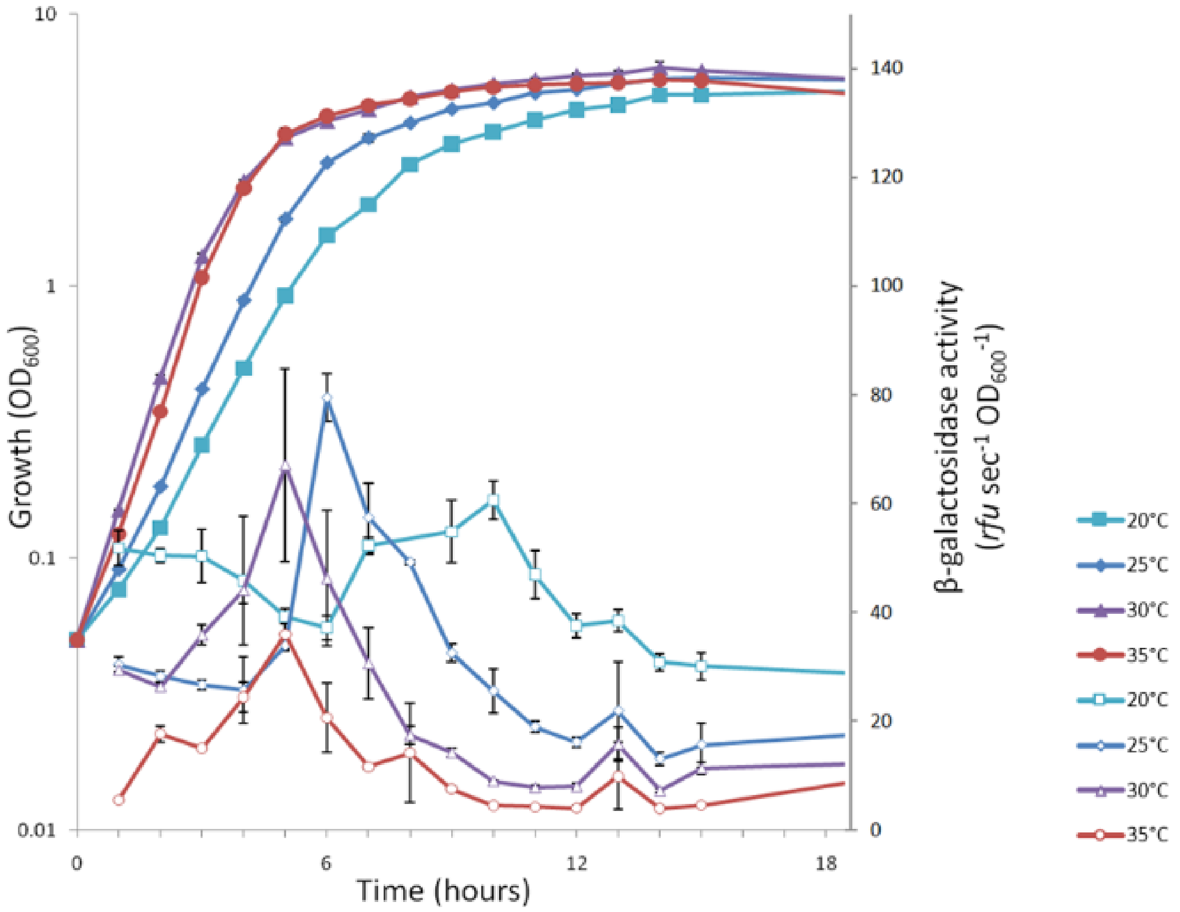

Figure S2. The expression of the *S. plymuthica* A153 *zmn* gene cluster is not temperature-dependent. Transcription of the *zmn* gene cluster in *Serratia plymuthica* A153 throughout growth at different temperatures. Data shown are the average values ± SD of at least three experiments.

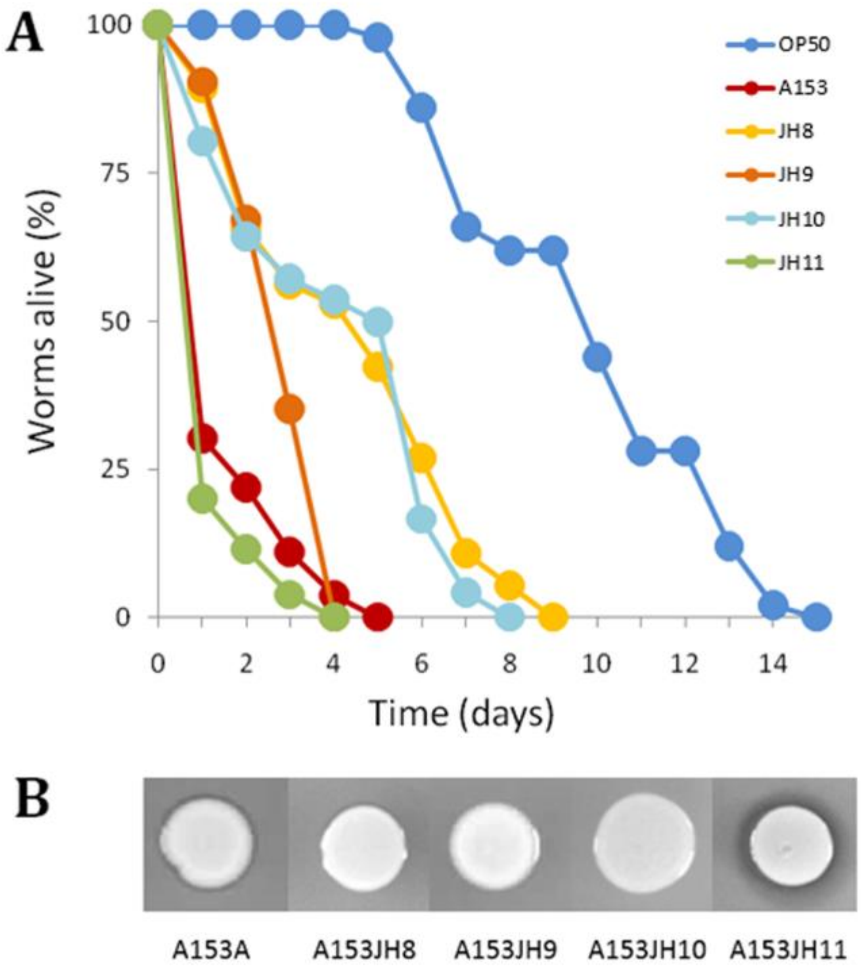

Figure S3. Kinetics of the nematicidal properties of *Serratia plymuthica* A153 strains. (A) Compared with the A153A parent strain, mutants A153JH8, A153JH9, and A153JH10, show reduced virulence against *C. elegans* ($P << 0.05$). In contrast, mutant A153JH11 shows increased zeamine production although this does not result in significantly increased virulence in *C. elegans* ($P = 0.12$). (B) Antibacterial phenotypes of the parent and mutant strains after an overnight incubation at 25°C.

1.3. Supplementary references

Bertani, I., da Silva, D. P., Abbruscato, P., Piffanelli, P., and Venturi, V. (2013). Draft genome sequence of the plant pathogen *Dickeya zeae* DZ2Q, isolated from rice in Italy. *Genome Announc.* 1, e00905-e00913. doi:10.1128/genomeA.00905-13

Cleto, S., Matos, S., Kluskens, L., and Vieira, M. J. (2012). Characterization of contaminants from a sanitized milk processing plant. *PLOS ONE*, 7: e40189. doi:10.1371/journal.pone.0040189

Hussain, M. B., Zhang, H. B., Xu, J. L., Liu, Q., Jiang, Z., and Zhang, L. H. (2008). The acyl-homoserine lactone-type quorum-sensing system modulates cell motility and virulence of *Erwinia chrysanthemi* pv. *zeae*. *J. Bacteriol.* 190, 1045-1053.

Li, B., Shi, Y., Ibrahim, M., Liu, H., Shan, C., Wang, Y., *et al.* (2012). Genome sequence of the rice pathogen *Dickeya zeae* strain ZJU1202. *J. Bacteriol.* 194, 4452-4453.

Van Houdt, R., Aertsen, A., Jansen, A., Quintana, A. L., and Michiels, C. W. (2004). Biofilm formation and cell-to-cell signalling in Gram-negative bacteria isolated from a food processing environment. *J. Appl. Microbiol.* 96, 177-184.
